# Feature-Based Molecular Networking Facilitates the Comprehensive Identification of Differential Metabolites in Diabetic Cognitive Dysfunction Rats

**DOI:** 10.3390/metabo13040538

**Published:** 2023-04-10

**Authors:** Ke Du, Chuanjia Zhai, Xuejiao Li, Hongchuan Gang, Xiaoyan Gao

**Affiliations:** School of Chinese Materia Medica, Beijing University of Chinese Medicine, Beijing102488, China

**Keywords:** feature-based molecular networking, metabolomics, diabetic recognition dysfunction, differential metabolites, comprehensive identification

## Abstract

Cognitive dysfunction is a frequent complication of type 2 diabetes mellitus (T2DM), usually accompanied by metabolic disorders. However, the metabolic changes in diabetic cognitive dysfunction (DCD) patients, especially compared to T2DM groups, are not fully understood. Due to the subtle differences in metabolic alterations between DCD groups and T2DM groups, the comprehensive detection of the untargeted metabolic profiles of hippocampus and urine samples of rats was conducted by LC–MS, considering the different ionization modes and polarities of the examined compounds, and feature-based molecular networking (FBMN) was performed to help identify differential metabolites from a comprehensive perspective in this study. In addition, an association analysis of the differential metabolites in hippocampus and urine was conducted by the O2PLS model. Finally, a total of 71 hippocampal tissue differential metabolites and 179 urine differential metabolites were identified. The pathway enrichment results showed that glutamine and glutamate metabolism, alanine, aspartate, and glutamate metabolism, glycerol phospholipid metabolism, TCA cycle, and arginine biosynthesis in the hippocampus of DCD animals were changed. Seven metabolites (AUC > 0.9) in urine appeared as key differential metabolites that might reflect metabolic changes in the target tissue of DCD rats. This study showed that FBMN facilitated the comprehensive identification of differential metabolites in DCD rats. The differential metabolites may suggest an underlying DCD and be considered as potential biomarkers for DCD. Large samples and clinical experiments are needed for the subsequent elucidation of the possible mechanisms leading to these alterations and the verification of potential biomarkers.

## 1. Introduction

In the wake of the widespread changes in lifestyle, diabetes is becoming more and more prevalent [1]. As a common complication of diabetes, diabetic cognitive dysfunction (DCD) has received increasing attention [2,3,4]. At a group level, people with DCD perform worse in the domains of information processing speed, attention and concentration, executive functions, and working memory, which negatively impacts their quality of life [5,6]. 

As is well known, diabetes is a worldwide metabolic disease [7], and the development of DCD is often accompanied by correlated metabolic changes [8,9]. In recent years, untargeted metabolomics has played a critical role in characterizing metabolic alterations and elucidating underlying mechanisms of neurodegenerative disease [10,11,12,13]. Nevertheless, metabolic disorders in DCD versus T2DM patients have been less extensively described. To address this, the comprehensive detection of metabolite features is essential, and the study methods should allow the analysis of the different polarities and MS ionization modes of molecules [14,15]. More importantly, it is a challenge to identify differential metabolites comprehensively based on a large amount of information.

Feature-based molecular networking (FBMN) has been from molecular networking combined with feature detection methods [16,17]. This workflow has achieved the discrimination of isomers by their retention time when creating molecular networking data and the evaluation of their compatibility with the in silico annotation of MS/MS spectra, reducing molecule redundancy and facilitating the annotation of metabolites by matching their spectra with those in mass spectral libraries and comparing them with those of similar compounds [16,18]. In recent years, FBMN has been frequently employed in the field of phytochemical composition and drug metabolism analyses [19,20,21], while it has been rarely applied in the identification of differential metabolites in disease states.

**Figure 1:**
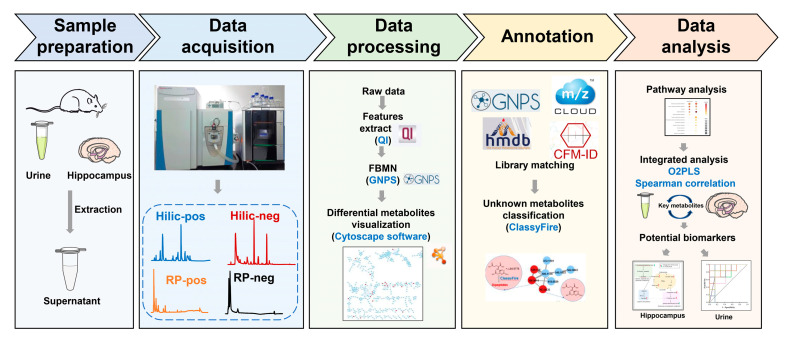
Overview of the study workflow.

The purpose of the present work was to comprehensively identify differential metabolites and characterize the metabolic alterations in DCD rats versus T2DM rats. To acquire comprehensive metabolic profiles of urine and hippocampal tissue of diabetic rats with cognitive dysfunction, positive and negative ion modes and reverse-phase (RP) and hydrophilic interaction chromatography (HILIC) columns were used in LC-MS analyses, respectively. Furthermore, FBMN was conducted to fully attribute the metabolites. The detailed workflow is shown in Figure 1. Finally, 71 differential metabolites in the hippocampus and 179 differential metabolites in the urine of DCD rats were identified. The pathway enrichment results showed that glutamine and glutamate metabolism, alanine, aspartate, and glutamate metabolism, glycerophospholipid metabolism, TCA cycle, arginine biosynthesis were changed in the hippocampus of DCD rats. Seven metabolites (AUC > 0.9) in urine appeared as key differential metabolites that might reflect metabolic changes in the target tissue of DCD. This work describes a strategy for identifying differential metabolites in DCD rats, providing a new view for metabolic disturbances in DCD.

## 2. Materials and Methods

### 2.1. Reagents and Materials

LC-MS-grade acetonitrile and formic acid were purchased from Thermo Fisher Scientific (Waltham, MA, USA). The water used for the LC-MS analysis was obtained from Watsons. The citric acid/sodium citrate buffer (Lot No. A20HR180485) and streptozocin (STZ, Lot No. O10GS163045) were purchased from Shanghai yuanye Bio-Technology (Shanghai, China).

### 2.2. Animal Treatment

Twenty-four male SD rats (6 weeks old, 180 ± 10 g, approval number: SCXK (Beijing, China) 2019-0010) were housed. The temperature was controlled at 21–25 °C, the relative humidity was 55–65%, and the cycle of day and night was 12 h. The rats adapted to the environment for 7 days. All experiments were approved by the Ethics Committee for Animal Care and Treatment at Beijing University of Chinese Medicine (BUCM-4-2022033002-1051).

**Figure 2:**
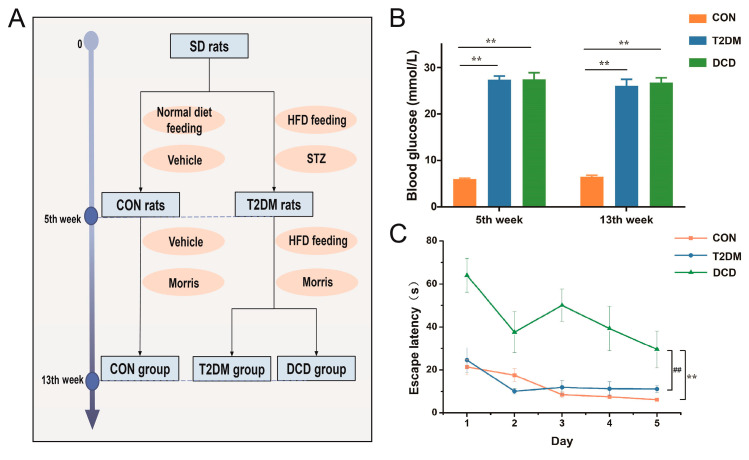
(**A**) Flow diagram of animal grouping and basic and behavior characteristics of the CON, T2DM, and DCD groups. (**B**) Random blood glucose levels; (**C**) average latency of the rats in the Morris water maze. (CON = control, T2DM = type 2 diabetes mellitus, DCD = diabetic cognitive dysfunction, ** *p* < 0.01, compared with CON group, ## *p* < 0.01 compared with T2DM group).

Eight rats were fed a normal diet, and 16 rats were fed a high-fat diet (HFD12491). After 5 weeks of HFD feeding, the rats were intraperitoneally injected with STZ (35 mg·kg^−1^) dissolved in the citric acid/sodium citrate buffer (0.01 M, pH 4.5), and the control (CON) group rats were injected with vehicle. Seventy-two hours after the STZ injection, the rats with a blood glucose value > 16.7 mmol·L^−1^ were recognized as diabetic rats and were selected for the subsequent experiments. At the 13th week, the rats were divided into a CON group, a T2DM group (whose escape latency was not statistically significant compared with that of the CON group), and a DCD group (whose escape latency was statistically significant compared with that of the CON group) by Morris water maze experiments. The flow diagram of animal grouping is shown in Figure 2A.

### 2.3. Morris Water Maze Test

The protocol of Morris water maze tests was as follows. The circular swimming pool used for the tests was 1.2 m in diameter. The water in the maze was made opaque by a non-toxic black dye and was maintained at 25 °C. The tank was divided into 4 equal quadrants, and the platform located in the third quadrant was immobilized 2 cm under the water surface. During the experiments, the light source intensity was constant, and unique geometric figures were placed at the sides of the pool to create visual clues for the rats. The rats were trained 4 times per day for 5 days. In each trail, the rat was gently released into the pool from 4 different quadrants. A maximum of 90 s was set for each rat to find the hidden platform. Once the rat found the platform, it was allowed to stay on it for 15 s. If the rat failed, it would be guided to the platform and allowed to stay on it for 15 s, and the escape latency of the rat was recorded as 90 s. The whole process of the experiment was traced by a video tracking system (Noldus Information Technology Co., Ltd., Wageningen, the Netherlands).

### 2.4. Sample Preparation

#### 2.4.1. Hippocampus Sample Preparation

Firstly, the hippocampal tissue was thawed at room temperature. Then, precooled methanol was added to a part of the hippocampal tissue to obtain a 20 mL·g^−1^ solution, and the sample was homogenized at 60 Hz for 120 s at 4 °C. After centrifuging at 13,600× *g* for 15 min at 4 °C, 650 μL of the supernatant was taken and dried in nitrogen. The residue was subsequently redissolved in 70 μL of 50% methanol and centrifuged at 13,600× *g* for 10 min at 4 °C. The supernatant was stored for the RP analysis. To monitor the stability of the Q-Exactive Orbitrap MS system, quality control (QC) samples, which were pooled form 10 μL of each sample, were detected after every eight samples. As for the HILIC analysis, another part of the hippocampal tissue was treated with precooled 50% methanol to obtain a 20 mL·g^−1^ solution. Then, the sample was homogenized at 60 Hz for 120 s at 4 °C and centrifuged at 13,600× *g* for 15 min at 4 °C. The supernatant was taken for subsequent analyses. The QC samples were prepared from 10 μL of each sample and injected after every eight samples.

#### 2.4.2. Urine Sample Preparation

For the RP analysis, after thawing at room temperature, 100 μL urine was diluted with 100 μL water and vortexed for 30 s. Then, the mixture was centrifuged at 13,600× *g* for 15 min at 4 °C, and the supernatant was collected for LC-MS analysis. The QC samples were obtained by mixing 10 μL of each sample. In addition, another 100 μL of urine was added to 400 μL of a methanol–acetonitrile solution (*v*/*v*, 1:1). Then, the mixture was vortexed for 30 s and centrifuged at 13,600× *g* for 15 min at 4 °C. The supernatant was prepared for the HILIC analysis. The QC samples were prepared by mixing 10 μL of each sample and lined at regular intervals (every 8 samples).

### 2.5. Data Acquisition for Untargeted Metabolomics Profiling

The UPLC analysis was conducted by a Vanquish UPLC system (Thermo Fisher Scientific, Waltham, MA, USA). The chromatographic conditions were as follows.

#### 2.5.1. Chromatographic Conditions for the hippocampus Samples

For the RP analysis, a HSS T3 column (2.1 mm × 100 mm, 1.8 μm, Waters Co., Milford, MA, USA) was used at 40 °C. The mobile phase consisted of 0.1% formic acid–water (*v*/*v*) (A) and 0.1% formic acid–acetonitrile (*v*/*v*) (B). The solvent program was set as follows: 0–1 min, 1% B; 1–4 min, 1–20% B; 4–6.5 min, 20–50% B; 6.5–8 min, 50–98% B; 8–10 min, 98% B; 10–10.1 min, 98–1% B; 10.1–13 min, 1% B. The flow rate was set at 0.4 mL·min^−1^, and the injection volume was 5 μL. For HILIC detection, a BEH amide column (2.1 mm × 100 mm, 1.7 µm, Waters Co., Milford, MA, USA) was used at 40 °C. The mobile phase A was composed of water with 10 mM ammonium formate and 0.125% formic acid, the mobile phase B was composed of acetonitrile modified by the addition of 10 mM ammonium formate and 0.125% formic acid and followed at a rate of 0.4 mL·min^−1^ with a 13 min gradient: 0–2 min, 95% B; 2–7.7 min, 95–70% B; 7.7–9.5 min, 70–40% B; 9.5–10.5 min, 40–30% B; 10.5–11 min, 30–95% B; 11–13 min, 95% B. The flow rate was set to 0.3 mL·min^−1^, and the injection volume was 3 μL.

#### 2.5.2. Chromatographic Conditions of the Urine Samples

For the RP analysis, the mobile phase A was 0.1% formic acid–water (*v*/*v*), while the mobile phase B was acetonitrile. The solvent gradient was set as follows: 0–4 min, 1% B; 4–12 min, 1–50% B; 12–16 min, 50–98% B; 16–18 min, 98% B; 18–18.1 min, 98–1% B; 18.1–20 min, 1% B. The oven temperature was kept at 40 °C, the flow rate was set to 0.3 mL·min^−1^ and the injection volume was 5 μL. For the HILIC analysis, the mobile phase A was composed of water with 10 mM ammonium formate and 0.1% formic acid (*v*/*v*), the mobile phase B was composed of acetonitrile/water (95/5, *v*/*v*) with 10 mM ammonium formate and 0.1% formic acid (*v*/*v*). A gradient run was set up as follows: 0–2 min, 95% B; 2–4.5 min, 95–85% B; 4–7.5 min, 85–75% B; 7.5–8.5 min, 75–70% B; 8.5–9.5 min, 70–40% B; 9.5–10 min, 40–30% B; 10–10.5 min, 30–95% B; 10.5–12 min, 95% B. The flow rate was kept at 0.3 mL min^−1^ with 40 °C, and the injection volume was 3 μL.

#### 2.5.3. MS Conditions

The MS parameters of both hippocampus samples and urine samples were similar. Data-dependent acquisition in ESI positive and negative ionization modes using the Q-Exactive Orbitrap MS (Thermo Fisher Scientific, Waltham, MA, USA) was performed. The acquisition settings were as follows: spray voltage, 3.5 kV or −2.8 kV; capillary temperature, 275 °C; auxiliary gas heater, 350 °C; sheath gas, 40 (Arb, arbitrary unit); auxiliary gas, 10 (Arb), and mass range, 70–1050 Da (hippocampus samples) or 60–900 Da (urine samples).

### 2.6. Metabolomics Data Analysis

The raw data were denoised, identified, aligned, and normalized by Progenesis QI software (Waters Co., Milford, MA, USA). The raw data were converted into a data matrix including t_R_-*m/z* ion pairs, sample names, and peak intensifiers and then imported into the multivariate statistical software SIMCA-P 14.0 (Umetrics AB, Umea, Sweden) to perform an orthogonal partial least-squared discriminant analysis (OPLS-DA) analysis. Besides, the data processed by QI software were be imported into the Global Natural Products Social Molecular Networking (GNPS) website (https://gnps.ucsd.edu/, accessed on 25 October 2022) for FBMN and visualized via Cytoscape software. In addition to GNPS, the HMDB database (http://www.hmdb.ca/, accessed on 30 October 2022), mzcloud (https://www.mzcloud.org/, accessed on 30 October 2022), CFM-ID 4.0 (https://cfmid.wishartlab.com/, accessed on 30 October 2022), and ClassyFire website (http://classyfire.wishartlab.com/, accessed on 5 November 2022) were used to characterize differential metabolites. Metabolic pathway analysis was performed using the MetaboAnalyst website (https://www.metaboanalyst.ca/, accessed on 15 November 2022) and KEGG database (https://www.genome.jp/kegg/, accessed on 15 November 2022). 

Statistical analysis was conducted on SPSS 22.0 (SPSS, IBM, New York, NY, USA). The statistical differences between two groups were compared by the Student’s t-test, and the statistical differences between multiple groups were compared by one-way ANOVA. Particularly, Student’s t-test and ANOVA analysis were conducted for normally distributed values, and the Mann–Whitney test was used for non-parametric values.

## 3. Results and Discussion

### 3.1. Establishment of the DCD Rat Model

T2DM was induced by intraperitoneal injection of STZ (35 mg·kg^−1^) in SD rats after 5 weeks of high-fat feeding. Then, the diabetic rats were fed a high-fat diet for another 8 weeks. Finally, 13 surviving diabetic rats were subjected to Morris water maze experiments, and 6 rats with cognitive impairment were screened out by escape latency analysis. As shown in Figure 2B,C, the levels of blood glucose in diabetic rats were higher compared with those of normal rats, and there was a significant difference in escape latency between the DCD group and the T2DM group (*p* < 0.01). The prolonged escape latency indicated a decline in cognitive ability in the DCD rats [9]. Finally, a total of six DCD rats were obtained, and the success rate was only ~46%.

### 3.2. Altered Metabolic Profiles of Hippocampal Tissue and Urine in Rats with DCD

**Figure 3:**
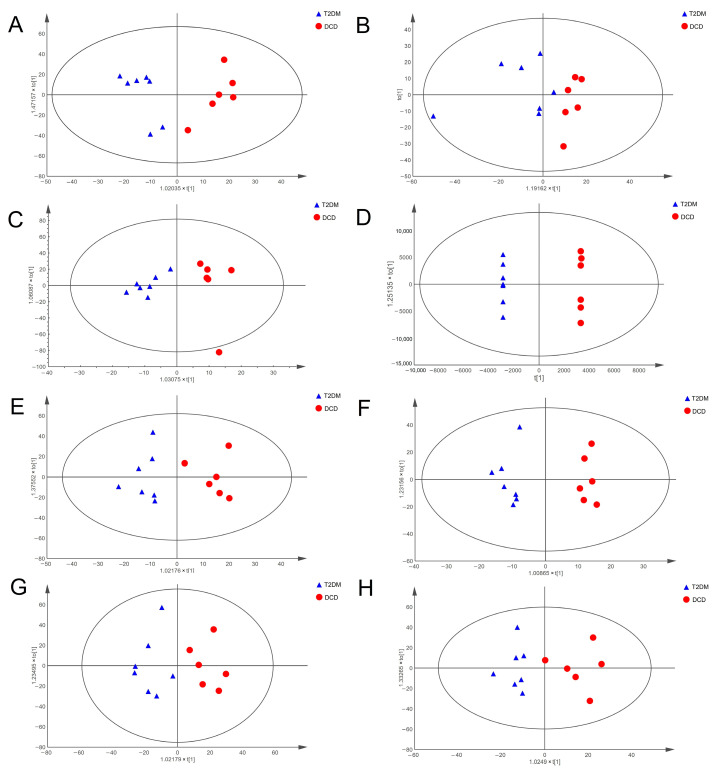
OPLS-DA models of hippocampal tissue and urine. OPLS-DA score plots of hippocampal tissues in positive (**A**) and negative ion modes (**B**) using the HILIC column; OPLS-DA score plots of hippocampal tissues in positive (**C**) and negative ion modes (**D**) using the RP column; OPLS-DA score plots of urine in positive (**E**) and negative ion modes (**F**) using the HILIC column; OPLS-DA score plots of urine in positive (**G**) and negative ion modes (**H**) using the RP column.

In order to obtain comprehensive metabolic profiles, hippocampus samples and urine samples were analyzed with HILIC and RP columns, respectively, and data using both positive and negative ion modes were collected. After preprocessing by the QI software, the metabolic profiles were analyzed using the OPLS-DA model. The results showed an obvious separation between the T2DM and DCD groups for both hippocampus samples and urine samples (Figure 3), indicating that diabetic cognitive dysfunction led to significant metabolic changes. 

### 3.3. Identification of Differential Metabolites Based on FBMN

**Figure 4:**
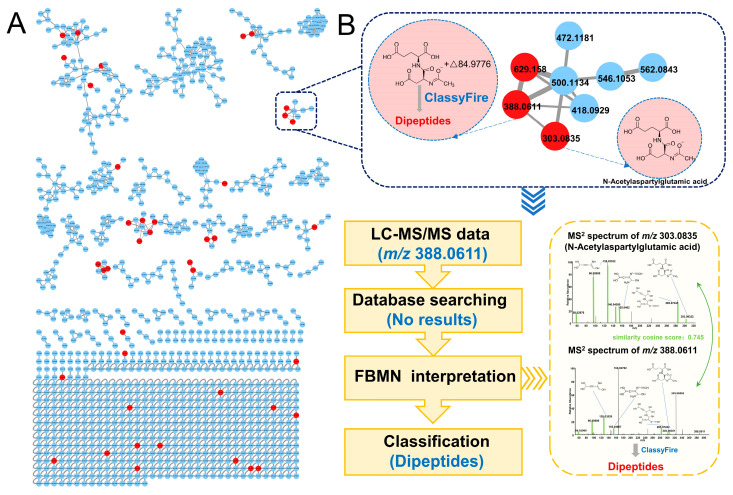
(**A**) FBMN of hippocampal tissue in negative ion mode using the RP column; (**B**) an example of annotation pipeline of the feature (*m/z* 388.0611) by FBMN. (The differential features are colored in red, the *m/z* of each feature is displayed at the center of the nodes, and the width of the edges show the magnitude of the cosine score, FBMN: feature-based molecular networking).

The VIP values of the OPLS-DA model were calculated, then the parameters VIP > 1 and *p* < 0.05 were used as a threshold to screen differential features. The feature data processed by QI were uploaded to the GNPS website. Based on the similarity principle of MS/MS spectrometry of features [22], FBMN was performed and subsequently visualized via Cytoscape software (Figure 4A, Supplementary Materials Figures S1–S4), where differential features were highlighted (red for differential features).

**Figure 5:**
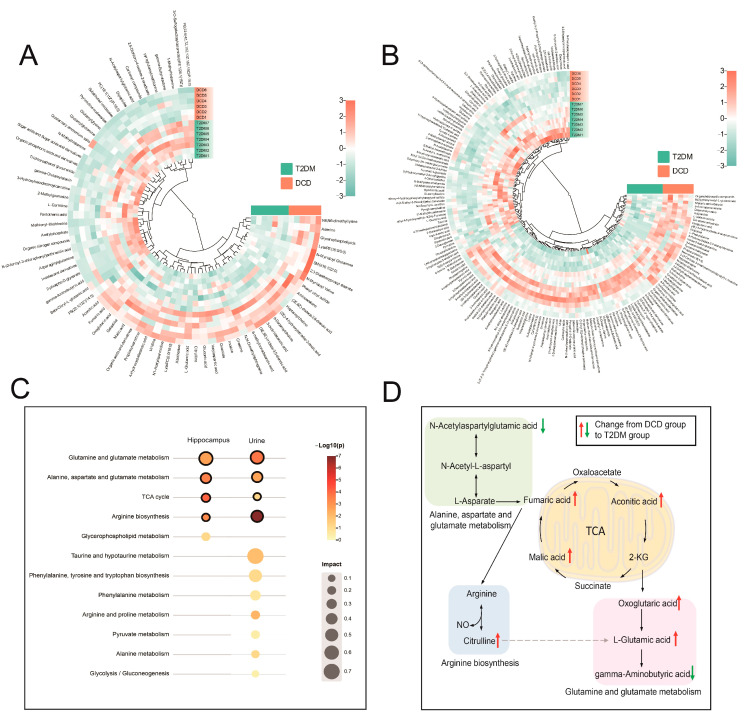
Heatmaps of differential metabolites in hippocampus (**A**) and urine samples (**B**); (**C**) metabolic pathway analysis of differential metabolites in hippocampal tissue and urine; (**D**) changes in the key pathways.

The richness and complexity of metabolite information make it difficult to annotate differential metabolites. For the sake of identifying differential metabolites, FBMN through the GNPS platform, HMDB database, and ClassyFire website was carried out. In addition to online matching known metabolites with mass spectral libraries through the GNPS platform, FBMN can also allow the attribution of unknown metabolites by comparison with known metabolites based on similarity cosine scoring of the MS/MS spectra. For example, as shown in Figure 4B, to annotate the unknown feature with *m/z* 388.0611, the adjacent known metabolite in FBMN (*m/z* 303.0835, similarity cosine value with the feature with *m/z* 388.0611 of 0.745) was firstly identified as N-acetylaspartylglutamic acid on the online database. Next, *m/z* 388.0611 was assigned to the class of dipeptides by ClassyFire according to their common characteristic fragments with *m/z* of 58.0298, 96.0091, 128.0353, 146.0459, 155.0462, 285.0725, 303.0832 [23,24]. Finally, 71 differential metabolites were identified in the hippocampus, among which No. 61–71 were classified by FBMN based on ClassyFire (Supplementary Materials Table S1); in addition, 179 urine differential metabolites were obtained, and No.166–179 were described using taxonomies or ontologies by FBMN and ClassyFire (Supplementary Materials Table S2). The clustering heat maps of the peak area of differential metabolites are shown in Figure 5A,B which represent the metabolic changes between DCD rats and T2DM rats visually. The ‘‘living data’’ concept of GNPS that the data can be continuously reanalyzed allows the identification of more metabolites [25,26]. As GNPS matures, these molecules will be identified, which allows investigating more differential metabolites characteristic of DCD.

### 3.4. Enrichment of the Metabolic Pathways

To identify the key metabolic pathways of DCD, the differential metabolites were imported to MetaboAnalyst for pathway enrichment. As shown in Figure 5C, glutamine and glutamate metabolism, alanine, aspartate, and glutamate metabolism, glycerophospholipid metabolism, TCA cycle, arginine biosynthesis, whose impact values were higher than 0.1, were enriched in the hippocampus. For urine, 11 metabolic pathways appeared as the key pathways, including taurine and hypotaurine metabolism, glutamine and glutamate metabolism, phenylalanine, tyrosine, and tryptophan biosynthesis, arginine biosynthesis, alanine, aspartate, and glutamate metabolism, phenylalanine metabolism, arginine and proline metabolism, pyruvate metabolism, alanine metabolism, TCA cycle, and glycolysis/gluconeogenesis. In contrast, as the target tissue of cognitive impairment, the hippocampal tissue is more intuitively the target of metabolic changes. In summary, glutamine and glutamate metabolism, alanine, aspartate, and glutamate metabolism, TCA cycle, arginine biosynthesis were the metabolic pathways identified in both hippocampus and urine, suggesting that there may be a potential relationship between hippocampus and urine. 

Glutamate is the main excitatory neurotransmitter for the activation of the N-methyl-D-aspartic acid receptor (NMDAR) [27]. When excessive glutamate is released or astrocyte dysfunction occurs, glutamate accumulates in the synaptic cleft or even spills over, resulting in the overactivation of extrasynaptic NMDAR, which may lead to synaptic plasticity impairment and excitotoxicity [28,29,30,31]. This study found elevated levels of glutamate and 2-oxoglutarate in the hippocampal tissue of DCD rats, suggesting that neurotoxicity and impaired synaptic plasticity caused by glutamate accumulation might lead to DCD. 

As is known to all, the TCA cycle plays an important role in the energy metabolism of organisms [32,33]. The carbon chain degradation products of sugars, fats, proteins, and nucleic acids will eventually enter the TCA cycle and will be discharged from the body as CO_2_ [34,35]. The metabolic intermediates of the TCA cycle are also critical precursors of compound biosynthesis [36,37], which are crucial for maintaining homeostasis in cells. In this study, the levels of malic acid, fumaric acid, and aconite acid increased significantly, demonstrating that the energy metabolism in DCD rats was disturbed.

Arginine metabolism is related to the development of AD [38,39,40]. Arginine is converted to citrulline with the generation of NO by the action of endothelial nitric oxide synthase (eNOS) [41,42]. Generally, increased levels of citrulline are accompanied by increased levels of NO [43]. Studies have shown that NO can act as a neurotransmitter, mediating excitatory amino acids and synaptic transmission, and the production and excessive release of NO can directly lead to neurotoxicity [44,45,46]. Elevated citrulline levels in the hippocampal tissue suggest that the diabetic cognitive impairment might be due to neurotoxicity mediated by NO.

### 3.5. Integrated Analysis of Key Differential Metabolites in the Hippocampus and Urine of Rats with DCD

**Figure 6:**
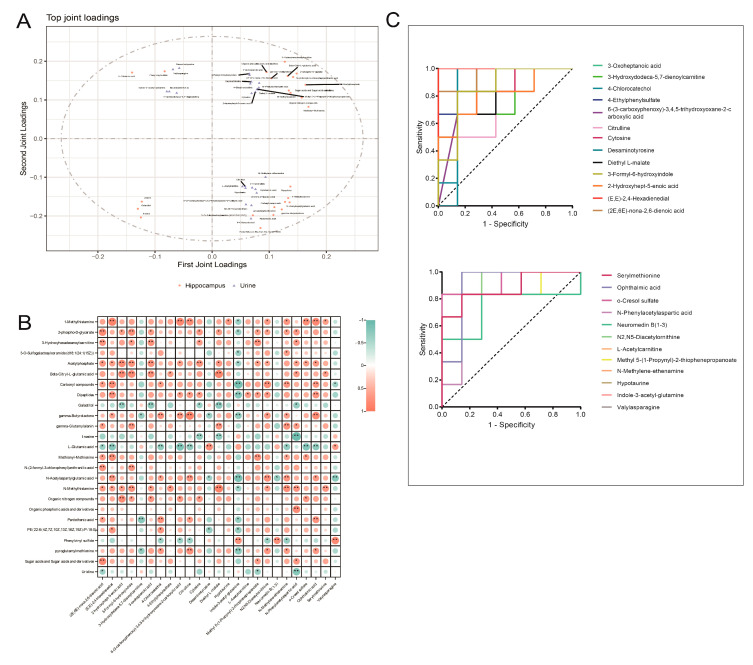
(**A**) Loading plot of the O2PLS model showing differential metabolites in hippocampal tissue and urine; (**B**) correlation plot illustrating the top 25 loading metabolites in hippocampal tissue and urine based on Spearman analysis, (the metabolites in urine are shown on the abscissa, and metabolites in the hippocampus on the ordinate, positive correlation is shown in orange, negative correlation in green, ** indicates *p*-value < 0.01 with |r| ≥ 0.6, * indicates *p*-value < 0.05 with |r| ≥ 0.6, and the size and depth of the circles indicate the magnitude of the correlation); (**C**) ROC analysis of the top 25 loading urine metabolites.

As the target tissue of cognitive dysfunction, metabolic alterations in the hippocampal tissue are of great significance to elucidate the underlying mechanism, while urine is the analyte of first choice for metabolomics research due to its easy access and abundant metabolites. In order to further explore the potential biomarkers of urine related to those in the hippocampal tissue in rats with DCD, a model of O2PLS was constructed. The model shows excellent performance in the integrated analysis of multiple metabolomics data, and its estimated values are close to the actual parameters in both low- and high-dimensional data [47]. The top 25 loading metabolites in urine and hippocampal tissue are shown in Figure 6A. To explore the relationship between the metabolites in hippocampal tissue and urine, Spearman correlation analysis with correlation coefficient ≥ 0.6 and *p* ≤ 0.05 as threshold was conducted. The results showed that the metabolites in the hippocampal tissue had a strong correlation with those in urine. For example, glutamate, which acts as a neurotransmitter in the brain [30], was negatively correlated with citrulline, ophthalmic acid, o-cresol sulfate, (2E,6E)-nona-2,6-dienoic acid, (E,E)-2,4-hexadienedial, N-methylene, and ethenamine and was positively correlated with desaminotyrosine and valylasparagine in urine. Inosine, which has been proven to display neuroprotective, anti-inflammatory, and antioxidant effects in the brain [48,49,50], was negatively correlated with cytosine, diethyl L-malate, and N-phenylaspartic acid in urine (shown in Figure 6B). This suggests that metabolic changes in urine may reflect those in the target tissues of DCD to a certain extent, and it is significant to illustrate the associations of metabolic alterations that occur in urine and hippocampal tissue.

The diagnostic value of urine metabolites associated with those of hippocampus was evaluated by the ROC curve (shown in Figure 6C). AUC > 0.9 was recognized as diagnostic. According to this standard, (E,E)-2,4-hexadienedial, (2E,6E)-nona-2,6-dienoic acid, o-cresol sulfate, ophthalmic acid, indole-3-acetyl-glutamine, N2,N5-diacetylornithine, and N-methylene ethenamine appeared as key differential metabolites that could reflect the metabolic changes in the hippocampus. However, due to the small number of samples, the capacity was limited, so clinical experiments with large samples should be conducted for subsequent verification.

With the prevalence of diabetes, DCD has attracted more and more attention. However, the issue of how to control DCD is still pending. Specifically, the molecular mechanisms of DCD have not been understood, and there is little information about the biomarkers of the disease. Since metabonomics research on the target sample of DCD is beneficial to uncover the molecular mechanisms of this dysfunction and the urine sample is the first choice to screen biomarkers, the analysis of differential metabolites in urine of DCD rats that could reflect the metabolic changes in the hippocampus is of great significance. The integration of metabonomics and FBMN will make the identification of differential metabolites more comprehensive and play an important role in the revelation of possible mechanisms and potential biomarkers.

## 4. Conclusions

Our study indicates that FBMN facilitates the identification of differential metabolites based on the comprehensive detection of untargeted metabolic profiles in DCD rats. Totally, 71 hippocampus differential metabolites and 179 urine differential metabolites in DCD rats were identified. Glutamine and glutamate metabolism, alanine, aspartate, and glutamate metabolism, TCA cycle, and arginine biosynthesis appeared as the critical pathways in the hippocampal tissue of DCD rats. Seven metabolites (AUC > 0.9) in urine appeared as key differential metabolites that might reflect metabolic changes in the target tissue in DCD rats. The identification and analysis of differential metabolites allowed the description of metabolic alterations in DCD rats, providing new insights for the diagnosis and molecular mechanism of this disease. Large samples and clinical experiments are needed for the subsequent elucidation of the possible mechanisms underlying this condition and the verification of potential biomarkers.

## Data Availability

The data presented in this study are available in the article.

## Supplementary Materials

The following supporting information can be downloaded at: https://www.mdpi.com/article/10.3390/metabo13040538/s1, Figure S1: FBMN of the hippocampal tissue by the HILIC column; Figure S2: FBMN of the hippocampal tissue in positive ion mode by the RP column; Figure S3: FBMN of urine by the HILIC column; Figure S4: FBMN of urine by the RP column; Table S1: Differential metabolites in hippocampal tissue; Table S2: Differential metabolites in urine.
