# Supplementary material for: Feature-Based Molecular Networking Facilitates the Comprehensive Identification of Differential Metabolites in Diabetic Cognitive Dysfunction Rats
Source: Metabolites. 2023 Apr 10;13(4):538. doi: 10.3390/metabo13040538 (PMC10142102; doi:10.3390/metabo13040538)
Supplement: Supplementary file 1 [file metabolites-13-00538-s001.zip › metabolites-2288937-supplementary.pdf]

metabolites

MDPI

Article

Feature-based Molecular Networking Facilitates Comprehensive Identification of Differential Metabolites in Diabetic Cognitive Dysfunction Rats

Ke Du, Chuanjia Zhai, Xuejiao Li, Hongchuan Gang, Xiaoyan Gao *

School of Chinese Materia Medica, Beijing University of Chinese Medicine, Beijing102488, China.
* Correspondence: gaoxiaoyan@bucm.edu.cn

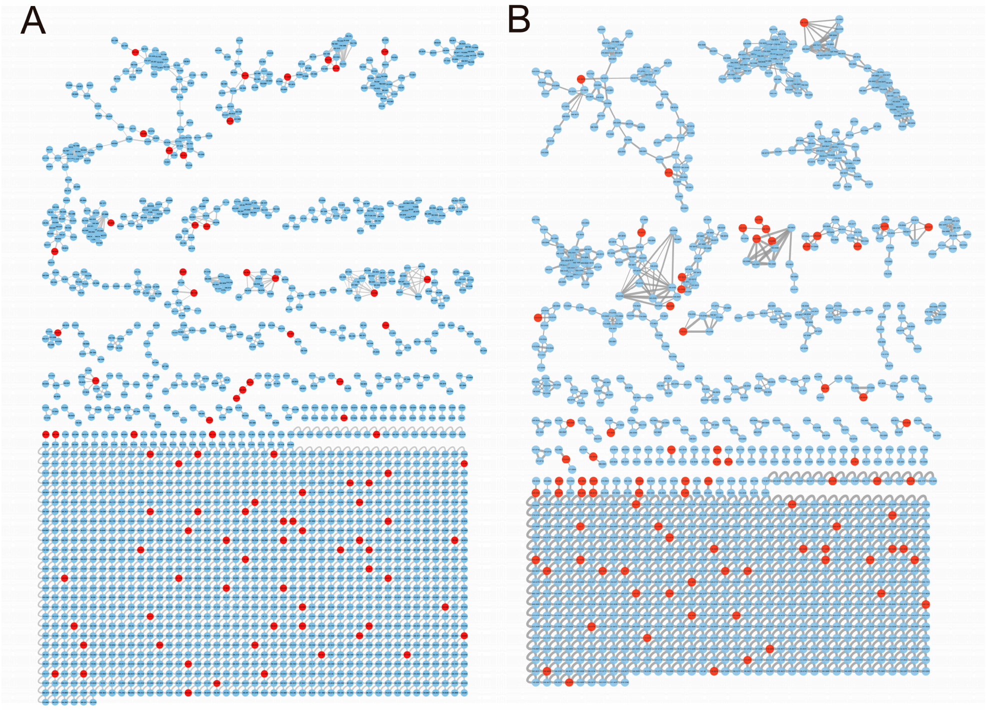

Figure S1. FBMN of the hippocampal tissue in positive (A) and negative (B) ion modes by the HILIC column. (The differential features are colored in red, the m/z of each feature is displayed at the center of the nodes, and the width of the edges show the magnitude of the cosine score, FBMN: feature-based molecular networking).

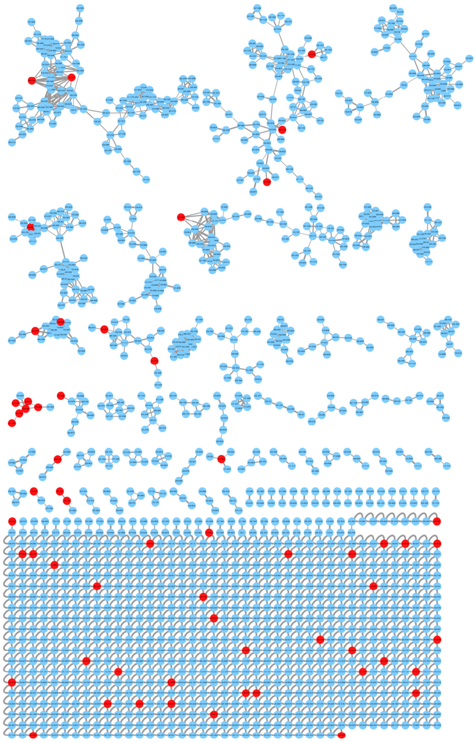

Figure S2. FBMN of the hippocampal tissue in positive ion mode by the RP column. (The differential features are colored in red, the m/z of each feature is displayed at the center of the nodes, and the width of the edges show the magnitude of the cosine score, FBMN: feature-based molecular networking).

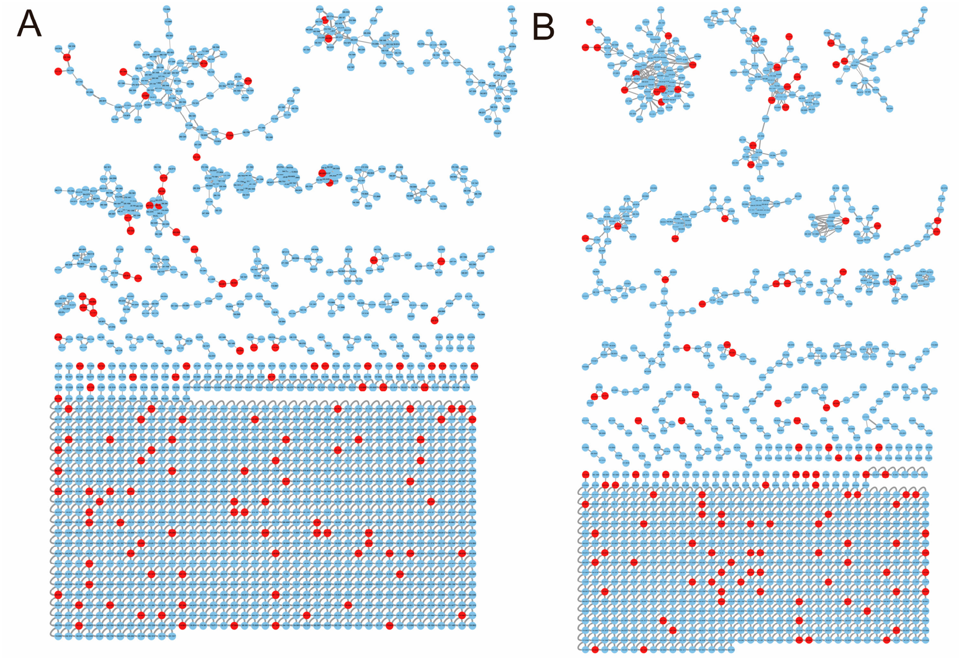

Figure S3. FBMN of urine in positive (**A**) and negative (**B**) ion modes by the HILIC column. (The differential features are colored in red, the m/z of each feature is displayed at the center of the nodes, and the width of the edges show the magnitude of the cosine score, FBMN: feature-based molecular networking).

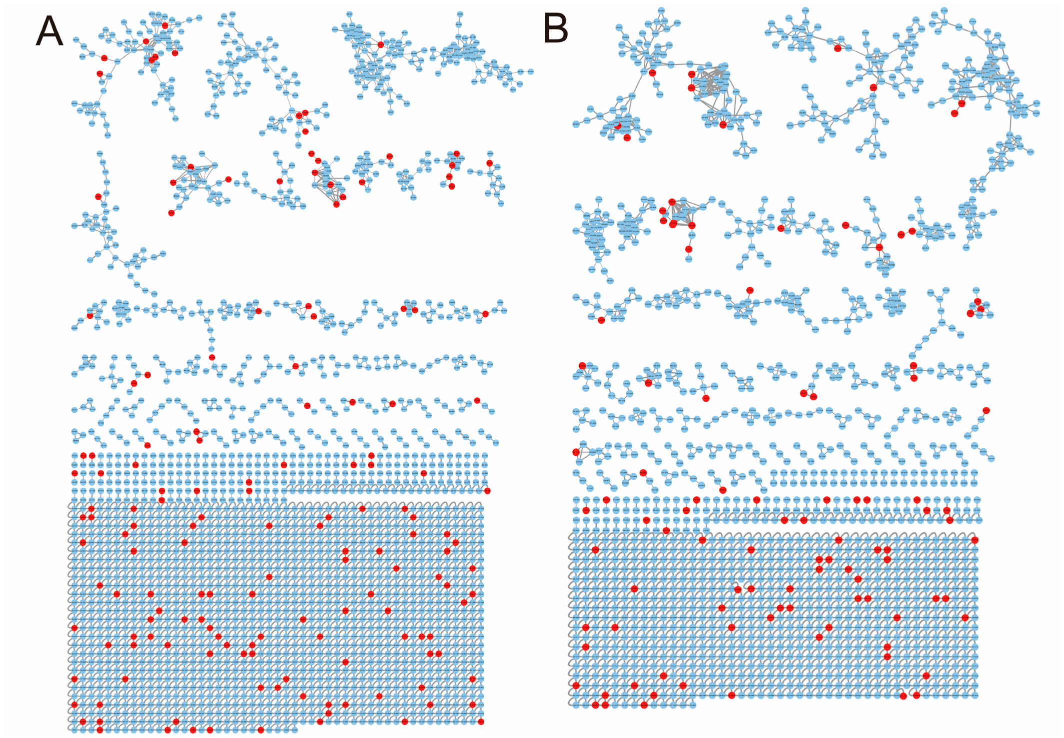

Figure S4. FBMN of urine in positive (**A**) and negative (**B**) ion modes by the RP column. (The differential features are colored in red, the *m/z* of each feature is displayed at the center of the nodes, and the width of the edges show the magnitude of the cosine score, FBMN: feature-based molecular networking).

Table S1. Differential metabolites in hippocampal tissues.

NO.	Metabolite/Class	HMDB	*m/z*	Formula	RT (min)	VIP	P value	LOG2FC (DCD/T2DM)
1	Aconitic acid	HMDB0000072	173.0088	C6H6O6	0.99	1.60	0.04	0.29
2	Inosine	HMDB0000195	267.0731	C10H12N4O5	3.25	1.37	0.01	0.24
3	N1-Acetylspermidine	HMDB0001276	188.1748	C9H21N3O	7.75	1.41	0.02	0.67
4	Aminoacetone	HMDB0002134	74.0600	C3H7NO	1.52	1.05	0.02	0.56
5	gamma-Glutamylalanin	HMDB0006248	217.0825	C8H14N2O5	5.89	1.63	0.04	-0.46
6	Trichloroethanol glucuronide	HMDB0042049	390.9373	C8H11Cl3O7	5.19	1.56	0.02	-1.38
7	2-phospho-D-glycerate	HMDB0304085	205.9607	C3H4O7P	4.67	1.59	0.01	-2.18
8	Adenine	HMDB0000034	136.0616	C5H5N5	4.10	1.47	0.03	0.23
9	L-Carnitine	HMDB0000062	162.1122	C7H15NO3	0.64	1.85	0.01	-0.58
10	Galactitol	HMDB0000107	181.0715	C6H14O6	5.50	1.12	0.04	0.47
11	gamma-Aminobutyric acid	HMDB0000112	104.0701	C4H9NO2	4.89	1.48	0.05	-2.16
12	Fumaric acid	HMDB0000134	115.0035	C4H4O4	7.99	1.67	0.01	0.24
13	L-Glutamic acid	HMDB0000148	148.0598	C5H9NO4	7.44	1.65	0.05	1.94
14	Malic acid	HMDB0000156	133.0140	C4H6O5	6.62	1.55	0.04	0.69
15	Oxoglutaric acid	HMDB0000208	145.0139	C5H6O5	0.99	1.68	0.02	0.42
16	Pantothenic acid	HMDB0000210	218.1034	C9H17NO5	3.58	3.25	0.02	-0.53
17	Uridine	HMDB0000296	243.0619	C9H12N2O6	2.03	1.42	0.02	0.31
18	Xanthosine	HMDB0000299	283.0684	C10H12N4O6	3.26	1.97	0.00	0.41
19	gamma-Butyrolactone	HMDB0000549	87.0439	C4H6O2	0.64	1.94	0.01	-0.41
20	Gluconic acid	HMDB0000625	195.0506	C6H12O7	7.53	1.52	0.02	0.67
21	Propionylcarnitine	HMDB0000824	217.1535	C10H19NO4	4.98	1.58	0.02	2.12
22	1-Methylhistamine	HMDB0000898	126.1024	C6H11N3	0.53	2.03	0.01	-1.71

23	Citrulline	HMDB0000904	176.1027	C6H13N3O3	0.99	1.59	0.02	0.52
24	N-Acetylaspartylglutamic acid	HMDB0001067	303.0835	C11H16N2O8	1.75	11.8	0.00	-0.82
25	Acetylphosphate	HMDB0001494	140.0104	C2H5O5P	7.81	1.50	0.02	-1.13
26	2-Methylguanosine	HMDB0005862	315.1409	C11H15N5O5	7.83	1.26	0.05	-1.46
27	Creatine	HMDB00064	130.0620	C4H9N3O2	6.57	1.49	0.02	0.17
28	PC(18:1(11Z)/P-18:0)	HMDB0008094	794.6009	C44H86NO7P	0.75	1.26	0.02	-1.74
29	PE(22:6(4Z,7Z,10Z,13Z,16Z,19Z)/P-18:0)	HMDB0009709	774.5480	C45H78NO7P	8.04	1.69	0.01	-0.47
30	LysoPC(0:0/18:0)	HMDB0011128	524.3709	C26H54NO7P	7.24	1.83	0.01	2.49
31	LysoPE(16:0/0:0)	HMDB0011503	454.2921	C20H40O2	7.68	1.62	0.04	0.45
32	3-O-Sulfogalactosylceramide (d18:1/24:1(15Z))	HMDB0012318	888.6276	C45H78NO7P	11.06	3.10	0.03	-0.56
33	9-Decenoylcholine	HMDB0013206	256.2265	C15H30NO2	7.34	2.28	0.00	0.57
34	Beta-Citryl-L-glutamic acid	HMDB0013220	320.0624	C11H15NO10	1.70	1.14	0.03	-0.55
35	Heptanoylcholine	HMDB0013239	216.1954	C12H26NO2	6.43	1.80	0.05	0.92
36	3-Hydroxyhexadecanoylcarnitine	HMDB0013336	416.3351	C23H45NO5	1.32	1.50	0.03	-1.14
37	N,N-Dimethylsphingosine	HMDB0013645	328.3201	C20H41NO2	7.49	1.37	0.03	0.95
38	Asparaginylglutamine	HMDB0028729	260.1340	C9H16N4O5	6.75	1.67	0.03	-0.60
39	Glutamylglycine	HMDB0028819	205.0811	C7H12N2O5	8.27	1.43	0.03	-1.11
40	Methionyl-Methionine	HMDB0028979	263.0882	C10H20N2O3S2	0.01	1.79	0.00	-2.52
41	Valylaspartic acid	HMDB0029123	231.0982	C9H16N2O5	7.49	1.03	0.04	0.44
42	Phenyl vinyl sulfide	HMDB0031825	137.0413	C8H8S	1.22	1.40	0.05	1.94
43	2,3-Diacetoxypropyl stearate	HMDB0059931	465.3182	C25H46O6	7.93	1.53	0.03	0.55
44	N-(2-formyl-3-chlorophenyl)anthranilic acid	HMDB0060006	298.0238	C14H10ClNO3	5.36	1.61	0.02	-1.93
45	2,5-Dichloro-4-oxohex-2-enedioate	HMDB0060363	226.9511	C6H4Cl2O5	0.55	1.90	0.01	-0.40
46	N-Methylhistamine	HMDB0061685	126.1020	C6H11N3	5.75	1.43	0.01	-1.53
47	PS(22:1(13Z)/14:0)	HMDB0112736	790.5565	C42H80NO10P	0.81	1.26	0.05	-0.59
48	SM(d16:1/22:0)	HMDB0240618	759.6348	C43H87N2O6P	7.93	1.58	0.01	0.95
49	N-Myristoyl Glutamine	HMDB0242050	730.5701	C19H36N2O4	7.98	1.48	0.00	0.95
50	N-Myristoyl Valine	HMDB0242063	327.2996	C19H37NO3	6.93	1.77	0.00	0.69
51	6-methylnonadecanoic acid	HMDB0340381	330.3357	C20H40O2	7.40	1.71	0.02	0.29
52	(2E,6Z)-dodeca-2,6-dienoic acid	HMDB0340680	214.1798	C12H20O2	6.69	2.08	0.02	0.97
53	4-Hydroxydodecanoic acid	HMDB0340733	216.1949	C12H24O3	0.75	1.56	0.03	1.26
54	(3E,5E)-trideca-3,5-dienoic acid	HMDB0340750	211.1689	C13H22O2	6.91	2.34	0.01	0.55
55	3-oxotridecanoic acid	HMDB0340773	228.1953	C13H24O3	6.91	2.36	0.00	0.56
56	(2E)-4-hydroxytetradec-2-enoic acid	HMDB0340788	242.2110	C14H26O3	7.13	2.15	0.00	0.44
57	N6,N6-dimethyllysine	HMDB0341183	174.1594	C8H18N2O2	2.32	1.25	0.05	1.03
58	Oxamide	HMDB0341188	88.0504	C2H4N2O2	5.63	1.24	0.03	1.49
59	pyroglutamylmethionine	HMDB0341384	259.0757	C10H16N2O4S	3.09	1.23	0.02	-0.68
60	Glutamylglutamine	HMDB28817	275.1336	C10H17N3O6	8.27	1.56	0.02	-0.83
61	Substituted imidazoles		136.8914		1.41	1.73	0.01	-0.89
62	Organic acids and derivatives		159.0102		6.62	1.56	0.05	0.67
63	Sugar acids and Sugar acids and derivatives		194.9056		3.65	1.12	0.01	-0.72
64	Pyrimidine nucleosides		243.9677		2.66	1.50	0.03	-0.58
65	Organic phosphoric acids and derivatives		244.9629		7.30	1.24	0.05	-0.35
66	Quaternary ammonium salts		146.9798		7.90	1.12	0.05	-0.99
67	Organic nitrogen compounds		166.0257		7.83	1.46	0.02	-0.74
68	Indoles and derivatives		205.1037		4.69	1.59	0.02	-3.50
69	Dipeptides		388.0611		1.79	2.77	0.00	-0.81
70	Carbonyl compounds		184.5503		1.65	2.23	0.00	-0.94
71	Glycerophospholipids		688.5183		8.31	1.96	0.01	0.66

Table S2. Differential metabolites in urine.

NO.	Metabolite/Class	HMDB	*m*/*z*	Formula	RT (min)	VIP	P Value	LOG2FC (DCD/T2DM)
1	1-Methylhistidine	HMDB0000001	170.0925	C7H11N3O2	2.14	1.35	0.03	0.81
2	4-Pyridoxic acid	HMDB0000017	206.0424	C8H9NO4	1.22	1.38	0.01	0.66
3	Dihydrobiopterin	HMDB0000038	239.1251	C9H13N5O3	2.42	1.12	0.02	-2.12
4	Dihydrouracil	HMDB0000076	173.0569	C4H6N2O2	1.15	1.50	0.02	1.15
5	Lysine	HMDB0000182	147.1129	C6H14N2O2	5.68	1.49	0.03	0.65
6	Ornithine	HMDB0000214	115.0867	C5H12N2O2	4.60	1.67	0.01	0.77
7	Urea	HMDB0000294	138.0980	CH4N2O	5.19	1.60	0.02	0.50
8	Glutamine	HMDB0000641	129.0659	C5H10N2O3	4.14	1.26	0.02	-0.50
9	Pelargonic acid	HMDB0000847	158.1541	C9H18O2	1.57	1.54	0.04	0.56
10	Indoleacetaldehyde	HMDB0001190	160.0758	C10H9NO	2.58	1.29	0.03	-0.94
11	Ophthalmic acid	HMDB0005765	289.1506	C11H19N3O6	7.54	1.61	0.03	-2.63
12	N-Acetylleucine	HMDB0011756	174.1126	C8 H15 N O3	4.07	1.59	0.04	-1.22
13	Propionylcholine	HMDB0013305	159.1494	C8H18NO2	1.79	1.34	0.03	2.26
14	Serylmethionine	HMDB0029045	237.0904	C8H16N2O4S	0.62	1.21	0.02	-1.98
15	Valylasparagine	HMDB0029122	232.1296	C9H17N3O4	2.94	1.37	0.03	1.95
16	Valylproline	HMDB0029135	215.1390	C10H18N2O3	0.88	1.13	0.03	-2.42
17	Tyrosine methylester	HMDB0029217	195.1116	C10H13NO3	4.87	1.52	0.03	2.19
18	Prenyl glucoside	HMDB0031876	307.1401	C11H20O6	9.92	1.45	0.00	0.95
19	Furaneol 4-glucoside	HMDB0032992	290.1232	C12H18O8	1.17	1.32	0.02	-0.93
20	Pyrraline	HMDB0033143	254.1499	C12H18N2O4	6.02	1.29	0.03	-0.64
21	11-hydroxyhexadeca-6,12-dienoic acid	HMDB0340891	269.2111	C16H28O3	12.20	1.26	0.03	-0.62
22	oct-5-enoic acid	HMDB0340975	141.0923	C8H14O2	11.38	1.47	0.02	0.54
23	2-hydroxyoct-4-enedioic acid	HMDB0340986	206.1024	C8H12O5	1.80	1.55	0.04	0.50
24	Adenosine	HMDB0000050	267.1206	C13H16N2O4	1.01	1.45	0.02	0.73
25	Glyceric acid	HMDB0000139	105.0194	C3H6O4	0.96	1.50	0.02	-0.80
26	L-Glutamic acid	HMDB0000148	130.0500	C5H9NO4	1.10	1.16	0.01	-0.24
27	L-Phenylalanine	HMDB0000159	166.0863	C9H11NO2	1.44	1.68	0.01	0.62
28	L-Acetylcarnitine	HMDB0000201	204.1232	C9H17NO4	4.66	1.31	0.04	-1.34
29	Oxoglutaric acid	HMDB0000208	129.0183	C5H6O5	1.17	1.22	0.05	0.61
30	Oxoadipic acid	HMDB0000225	159.0299	C6H8O5	5.77	1.27	0.04	0.53
31	Pyruvic acid	HMDB0000243	87.0087	C3H4O3	2.49	1.48	0.03	1.99
32	Taurine	HMDB0000251	124.0075	C2H7NO3S	1.39	1.34	0.01	1.45
33	3-Amino-2-piperidone	HMDB0000323	132.1132	C5H10N2O	3.31	1.55	0.03	0.58
34	Aminoadipic acid	HMDB0000510	160.0616	C6H11NO4	4.75	1.51	0.02	0.95
35	2-Octenedioic acid	HMDB0000341	171.0665	C8H12O4	10.11	1.41	0.01	0.46
36	3-Methoxy-4-hydroxyphenylethyleneglycol sulfate	HMDB0000559	263.0230	C9H12O7S	1.24	1.70	0.00	-1.48
37	Cytosine	HMDB0000630	112.0506	C4H5N3O	2.14	1.30	0.04	-0.44
38	Glucaric acid	HMDB0000663	209.0304	C6H10O8	0.94	1.58	0.03	-1.20
39	L-Leucine	HMDB0000687	114.0914	C6H13NO2	10.09	1.62	0.02	0.79
40	L-2-Hydroxyglutaric acid	HMDB0000694	147.0299	C5H8O5	7.02	1.57	0.00	0.52
41	5-Hydroxyindoleacetic acid	HMDB0000763	174.0563	C10H9NO3	6.48	1.36	0.02	-0.89
42	Vinylacetylglycine	HMDB0000894	124.0404	C6H9NO3	11.30	1.48	0.02	0.86
43	Citrulline	HMDB0000904	176.1031	C6H13N3O3	7.50	1.45	0.04	-1.34
44	Hypotaurine	HMDB0000965	110.0271	C2H7NO2S	6.48	1.26	0.03	-0.83
45	Dihydrolipoamide	HMDB0000985	225.1095	C8H17NOS2	2.73	1.41	0.03	-1.31
46	2-Aminobenzoic acid	HMDB0001123	138.0550	C7H7NO2	0.76	1.39	0.05	0.98
47	Nornicotine	HMDB0001126	166.1342	C9H12N2	18.79	1.42	0.05	0.40
48	4-Aminophenol	HMDB0001169	110.0601	C6H7NO	1.89	1.18	0.04	-0.31
49	5'-Methylthioadenosine	HMDB0001173	278.0704	C11H15N5O3S	7.55	1.09	0.02	0.52
50	N2-Succinyl-L-ornithin	HMDB0001199	231.0986	C9H16N2O5	7.06	1.42	0.04	-0.98

51	3,4-Dihydroxybenzeneacetic acid	HMDB0001336	151.0391	C8H8O4	0.22	1.48	0.02	0.63
52	4-Trimethylammoniobutanal	HMDB0001345	130.1227	C7H16NO	2.44	1.14	0.04	-2.24
53	N-Acetylputrescine	HMDB0002064	131.1180	C6H14N2O	0.97	1.47	0.00	0.64
54	Desaminotyrosine	HMDB0002199	225.0769	C9H10O3	1.01	1.44	0.03	0.45
55	Deoxyribose	HMDB0003224	133.0507	C5 H10 O4	1.75	1.59	0.00	-1.23
56	Pyrimidine	HMDB0003361	98.0715	C4H4N2	18.83	1.43	0.05	0.41
57	Formylanthranilic acid	HMDB0004089	148.0395	C8H7NO3	2.78	1.58	0.02	0.85
58	N1-Methyl-4-pyridone-3-carboxamide	HMDB0004194	135.0554	C7H8N2O2	9.83	1.79	0.00	1.39
59	Phytosphingosine	HMDB0004610	318.3011	C18H39NO3	16.60	1.26	0.04	0.46
60	Indolylacryloylglycine	HMDB0006005	243.0776	C13H12N2O3	11.57	1.22	0.05	1.13
61	N-Acetylglutamine	HMDB0006029	188.1031	C7H12N2O4	1.17	1.37	0.01	1.26
62	Putreanine	HMDB0006078	161.1286	C7H17N2O2	8.27	1.74	0.00	-0.58
63	Heptadecanoyl carnitine	HMDB0006210	431.3845	C24H47NO4	10.22	1.66	0.01	0.96
64	6'-Sialyllactose	HMDB0006569	632.2064	C23H39NO19	1.04	1.32	0.04	-1.23
65	Beta-D-Glucopyranuronic acid	HMDB0010314	373.0782	C13H14O9	7.72	1.47	0.04	-0.64
66	Malonic semialdehyde	HMDB0011111	147.0301	C3H4O3	1.51	1.62	0.03	-0.69
67	Aspartylhydroxyproline	HMDB0011160	245.0779	C9H14N2O6	1.49	1.24	0.04	0.94
68	2-(3-Carboxy-3-(methylammonio)propyl)-L-histidine	HMDB0011654	271.1401	C11H19N4O4	1.77	1.54	0.02	1.90
69	Cytidine 2',3'-cyclic phosphate	HMDB0011691	306.0497	C9H12N3O7P	19.08	1.45	0.05	0.51
70	N-Acetyl-L-methionine	HMDB0011745	190.0544	C7H13NO3S	1.46	1.23	0.04	-1.13
71	Cer(d18:0/14:0)	HMDB0011759	512.5040	C32H65NO3	0.70	1.65	0.01	1.21
72	5-Aminopentanamide	HMDB0012176	117.1023	C5H12N2O	4.62	1.60	0.03	0.76
73	Histidinal	HMDB0012234	140.0819	C6H9N3O	1.06	1.38	0.03	0.81
74	Isopentenyladenine-9-N-glucoside	HMDB0012240	383.2038	C16H23N5O5	7.33	1.40	0.04	-1.03
75	Methacholine	HMDB0015654	160.1333	C10H9NO	3.23	1.55	0.04	0.96
76	Alanylproline	HMDB0028695	187.1079	C8H14N2O3	5.56	1.59	0.02	0.84
77	Glutamylleucine	HMDB0028823	261.1446	C11H20N2O5	7.54	1.63	0.02	-1.84
78	Hydroxyprolyl-Proline	HMDB0028871	229.1184	C10H16N2O4	7.46	1.55	0.03	-1.95
79	Threonylhistidine	HMDB0029063	257.1243	C10H16N4O4	1.16	1.44	0.03	-0.56
80	3-Hydroxy-2-methylglutaric acid	HMDB0029169	161.0456	C6H10O5	2.26	1.20	0.04	0.42
81	N-Phenylacetylaspartic acid	HMDB0029355	250.0722	C12H13NO5	1.75	1.25	0.02	-1.24
82	Chymopapain	HMDB0029845	268.9433	C6H6O8S2	0.82	1.61	0.01	-1.68
83	2-Dodecylbenzenesulfonic acid	HMDB0031031	325.1846	C18H30O3S	1.75	1.58	0.01	0.53
84	3-Formyl-6-hydroxyindole	HMDB0031172	162.0550	C9H7NO2	0.97	1.02	0.03	-1.76
85	(E,E)-2,4-Hexadienedial	HMDB0031180	109.0295	C6H6O2	6.20	1.42	0.00	0.53
86	(2E,6E)-nona-2,6-dienoic acid	HMDB0031302	153.0923	C9H14O2	11.93	1.17	0.03	0.56
87	(E)-8-Hydroxy-2-octene-4,6-diynoic acid	HMDB0031515	151.0391	C8H6O3	19.06	1.43	0.03	0.61
88	Pipericine	HMDB0031678	336.3265	C22H41NO	18.25	1.32	0.04	0.43
89	O-Ethyl S,S-diphenyl phosphorodithioate	HMDB0031781	619.0400	C14H15O2PS2	1.69	1.41	0.03	-1.09
90	2,5-Dimethyl-3-furanthiol acetate	HMDB0032234	193.0288	C8H10O2S	19.08	1.46	0.02	0.60
91	N-Lactoyl ethanolamine	HMDB0032356	134.0813	C5H11NO3	10.05	1.51	0.05	0.61
92	2-(4-Methyl-5-thiazolyl)ethyl butanoate	HMDB0032418	444.1964	C10H15NO2S	2.22	1.50	0.01	-1.24
93	4-Pentenal	HMDB0032458	85.0650	C5H8O	17.85	1.46	0.05	0.40
94	N-Undecylbenzenesulfonic acid	HMDB0032549	311.1690	C17H28O3S	1.75	1.74	0.00	0.46
95	N-Carbamoylputrescine	HMDB0033458	132.1132	C5H13N3O	5.76	1.71	0.00	0.99
96	2,5-Undecadienal	HMDB0033546	189.1246	C11H18O	5.84	1.38	0.02	-1.02
97	2-Methyl-1-propylamine	HMDB0034198	74.0965	C4H11N	0.05	1.46	0.02	0.54
98	5-Hydroxymethyl-2-furancarboxaldehyde	HMDB0034355	125.0244	C6H6O3	7.00	1.41	0.05	0.64
99	Methyl 5-(1-Propynyl)-2-thiophenepropanoate	HMDB0034759	226.0897	C11H12O2S	0.95	1.31	0.02	-1.00

100	Xanthopurpurin	HMDB0036006	223.0393	C14H8O4	19.08	1.54	0.03	0.79
101	Ethyl 3,4,5-trimethoxybenzoate	HMDB0038627	239.0927	C12H16O5	11.89	1.32	0.05	0.60
102	S-Methyl hexanethioate	HMDB0039465	205.0905	C7H14OS	11.80	1.66	0.01	0.96
103	Diethyl L-malate	HMDB0040220	189.0770	C8H14O5	8.43	1.31	0.04	0.41
104	4-Methylbenzyl alcohol	HMDB0041609	121.0659	C5H4O3	11.72	1.62	0.00	1.02
105	Tyrosol 4-sulfate	HMDB0041785	217.0177	C8H10O5S	7.72	1.51	0.01	0.93
106	4-Chlorocatechol	HMDB0041810	166.9864	C6H5ClO2	0.53	1.51	0.01	-1.57
107	Pipemidic acid	HMDB0041989	321.1657	C14H17N5O3	7.48	1.45	0.01	-2.54
108	3-Acetamidobutanal	HMDB0059649	259.1652	C6H11NO2	1.85	1.45	0.01	1.67
109	2-Deoxypentonic acid	HMDB0059753	149.0455	C5H10O5	4.64	1.30	0.04	0.60
110	1,N2-propanodeoxyguanosine	HMDB0059780	288.1090	C13H17N5O4	1.87	1.51	0.01	1.17
111	Pyrogallol-2-O-sulphate	HMDB0060018	204.9812	C6H6O6S	1.31	1.48	0.02	0.85
112	(S)-3-Sulfonatolactate	HMDB0060176	168.9813	C3H6O6S	1.11	1.51	0.04	-1.42
113	2-aminophenol sulphate	HMDB0061116	190.0169	C6H7NO4S	0.74	1.76	0.00	-0.93
114	3-Hydroxyhexadecanoic acid	HMDB0061658	290.2694	C16H32O3	14.76	1.45	0.02	0.72
115	N-Methylene-ethenamine	HMDB0061870	111.0919	C3H5N	3.32	1.68	0.01	-3.73
116	Neuromedin B(1-3)	HMDB0013016	285.1569	C12H22N4O5	5.00	1.45	0.04	1.55
117	N-lactoyl-Methionine	HMDB0062182	222.0798	C8H15NO4S	2.82	1.34	0.04	1.83
118	2-Deoxyglucose	HMDB0062477	163.0614	C6H12O5	1.41	1.09	0.03	0.31
119	4-Ethylphenylsulfate	HMDB0062551	201.0228	C8 H10 O4 S	0.80	1.16	0.03	-1.03
120	N(2)-phenylacetyl-L-glutaminate	HMDB0062645	265.1183	C13H16N2O4	2.40	1.26	0.00	1.41
121	MG(i-14:0/0:0/0:0)	HMDB0072867	302.2698	C17H34O4	16.72	1.58	0.02	0.53
122	Pyroglutamylvaline	HMDB0094651	229.1183	C10H16N2O4	0.95	1.44	0.05	-1.04
123	N-Propionylalanine	HMDB0094698	146.0813	C6H11NO3	1.08	1.78	0.01	0.54
124	2-hydroxyhexanoylglycine	HMDB009471	188.0929	C8H15NO4	2.73	1.63	0.00	-1.47
125	6-(3-carboxyphenoxy)-3,4,5-trihydroxyoxane-2-carboxylic acid	HMDB0124994	332.0978	C13H14O9	7.40	1.33	0.03	-4.57
126	4-Methoxy-3-(sulfooxy)benzoic acid	HMDB0140929	246.9919	C8H8O7S	1.14	1.62	0.01	0.84
127	Hydroquinone sulfate	HMDB0240263	188.9865	C6H6O5S	6.20	1.47	0.00	0.82
128	N2,N5-Diacetylornithine	HMDB0240345	217.1186	C9H16N2O4	2.88	1.43	0.03	-1.93
129	Protocatechuic acid 4-O-sulfate	HMDB0240382	232.9763	C7H6O7S	1.11	1.37	0.03	0.75
130	5-(Hydroxymethyl-2-furoyl)glycine	HMDB0240476	198.0409	C8H9NO5	7.68	1.01	0.04	0.70
131	Hydroxytyrosol 4'-sulfate	HMDB0240532	233.0126	C8H10O6S	7.32	1.35	0.02	0.89
132	o-Cresol sulfate	HMDB0240655	247.0282	C7H8O4S	8.62	1.40	0.02	1.61
133	3-Methylcatechol 2-sulfate	HMDB0240663	203.0021	C7H8O5S	8.49	1.26	0.01	0.92
134	5-amino valeric acid betaine	HMDB0240732	142.1231	C8H17NO2	18.79	1.47	0.04	0.41
135	(4E)-3-Hydroxydodec-4-enoylcarnitine	HMDB0241220	299.1854	C19H35NO5	0.95	1.29	0.02	0.44
136	3-Hydroxydodeca-5,7-dienoylcarnitine	HMDB0241247	297.1695	C19H33NO5	0.97	1.23	0.03	0.22
137	2,5-Bis(acetylamino)pentanoic acid	HMDB0242108	215.1038	C9H16N2O4	4.49	1.74	0.00	-1.66
138	N(5)-Acetylornithine	HMDB0242109	157.0973	C7H14N2O3	4.54	1.55	0.02	0.45
139	N-Oleoyl-L-Serine	HMDB0242185	370.2958	C21H39NO4	18.10	1.47	0.03	0.44
140	13-Docosenamide	HMDB0244507	338.3422	C22H43NO	18.56	1.47	0.03	0.49
141	Citraconic anhydride	HMDB0250302	111.0089	C5H4O3	1.59	1.30	0.03	-0.37
142	L-isoleucinamide	HMDB0253897	131.1180	C6H14N2O	4.72	1.92	0.00	0.73
143	Succinamic acid	HMDB0258538	116.0353	C4H7NO3	1.69	1.52	0.01	0.64
144	ethyl-4-hydroxymethyl- 3(2H)-Furanone	HMDB0303864	283.1189	C7H10O3	11.53	1.33	0.00	0.42
145	indole-3-acetyl-glutamate	HMDB0304379	361.1046	C15H14N2O5	1.42	1.39	0.04	-2.98
146	indole-3-acetyl-glutamine	HMDB0304380	325.1023	C15H16N3O	2.33	1.52	0.01	2.14
147	Adipamide	HMDB0304762	127.0866	C6H12N2O2	1.08	1.70	0.02	0.36
148	4-Hydroxyphenylacetic acid sulfate	HMDB0304906	230.9969	C8H8O6S	8.64	1.36	0.01	0.79

149	2,3- Dimethoxyphenol sulfate	HMDB0304926	233.0126	C8H10O6S	0.95	1.33	0.05	-0.62
150	3-methylundecanoic acid	HMDB0340306	218.2115	C12H24O2	11.10	1.31	0.05	0.95
151	9-methyltridecanoic acid	HMDB0340321	246.2430	C14H28O2	14.49	1.47	0.02	0.89
152	4-methylpentadecanoic acid	HMDB0340340	274.2748	C16H32O2	16.57	1.34	0.03	0.50
153	6-methylnonadecanoic acid	HMDB0340381	312.3261	C20H40O2	10.24	1.53	0.04	0.59
154	5-hydroxydec-3-enedioic acid	HMDB0340543	215.0927	C10H16O5	10.13	1.64	0.00	0.46
155	dodeca-2,4-dienedioic acid	HMDB0340706	285.1346	C12H18O4	1.24	1.43	0.03	0.32
156	(2E,4Z)-hexadeca-2,4-dienedioic acid	HMDB0340897	283.1911	C16H26O4	0.72	1.52	0.04	0.60
157	hept-4-enoic acid	HMDB0340960	127.0766	C7H12O2	10.11	1.58	0.00	0.56
158	2-hydroxyhept-5-enoic acid	HMDB0340964	143.0715	C7H12O3	8.89	1.38	0.05	1.20
159	3-oxoheptanoic acid	HMDB0340970	203.0926	C7H12O3	9.78	1.08	0.03	0.75
160	5-hydroxyoct-2-enedioic acid	HMDB0340987	187.0612	C8H12O5	1.73	1.51	0.01	-1.09
161	non-6-enoic acid	HMDB0341014	155.1080	C9H16O2	12.56	1.51	0.03	0.87
162	(2E,6E)-nona-2,6-dienoic acid	HMDB0341032	153.0923	C9H14O2	11.93	1.17	0.03	0.56
163	6-Amino-1-MethylUracil	HMDB0341121	281.0993	C5H7N3O2	1.54	1.37	0.02	0.48
164	Dihydroxy-2H-indole glucuronide	HMDB0341439	324.0728	C14H15NO8	1.42	1.40	0.02	-2.01
165	5-(3',4',5'-Trihydroxyphenyl)-gamma-valerolactone-3'-O-glucuronide	HMDB59986	399.0938	C17H20O11	8.87	1.57	0.03	-0.85
166	Carboxylic acids		128.9596		5.44	1.39	0.05	0.39
167	Medium-chain keto acids and derivatives		157.8720		0.95	1.54	0.02	1.07
168	Amino acids and derivatives		147.0255		4.25	1.50	0.04	0.42
169	Dialkylamines		171.0958		7.54	1.55	0.05	-0.95
170	Hydroxybenzoic acid derivatives		139.5314		4.14	1.47	0.04	0.78
171	Imidazopyrimidines		166.9962		6.20	1.47	0.01	0.86
172	Lipids and lipid-like molecules		330.3012		17.06	1.50	0.03	0.54
173	Lipids and lipid-like molecules		762.5588		19.00	1.49	0.02	0.47
174	Medium-chain hydroxy acids and derivatives		159.8695		0.95	1.53	0.02	1.05
175	Organic acids and derivatives		296.9801		1.01	1.52	0.02	1.05
176	Organic acids and derivatives		468.4423		17.72	1.56	0.03	0.45
177	Organoheterocyclic compounds		255.0625		1.31	1.44	0.02	1.38
178	Organosulfur compounds		178.8805		0.91	1.58	0.02	1.10
179	Phenylpropanoic acids		164.8361		4.45	1.64	0.00	0.86
